# Characterization of Desulfurized Crumb Rubber/Styrene–Butadiene–Styrene Composite Modified Asphalt Based on Rheological Properties

**DOI:** 10.3390/ma14143780

**Published:** 2021-07-06

**Authors:** Jingyao Yang, Gang Xu, Peipei Kong, Xianhua Chen

**Affiliations:** School of Transportation, Southeast University, Nanjing 211189, China; yangjingyao19@foxmail.com (J.Y.); xugang619@hotmail.com (G.X.); kppwwy@163.com (P.K.)

**Keywords:** CR/SBS modified asphalt, desulfurized rubber, rheology, Burgers’ model, multiple stress creep recovery

## Abstract

With the growing interest in bituminous construction materials, desulfurized crumb rubber (CR)/styrene–butadiene–styrene (SBS) modified asphalts have been investigated by many researchers as low-cost environmental-friendly road construction materials. This study aimed to investigate the rheological properties of desulfurized CR/SBS composite modified asphalt within various temperature ranges. Bending beam rheometer (BBR), linear amplitude sweep (LAS), and multiple stress creep recovery (MSCR) tests were performed on conventional CR/SBS composite modified asphalt and five types of desulfurized CR/SBS modified asphalts. Meanwhile, Burgers’ model and the Kelvin–Voigt model were used to derive nonlinear viscoelastic parameters and analyze the viscoelastic mechanical behavior of the asphalts. The experimental results indicate that both the desulfurized CR/SBS composite modifier and force chemical reactor technique can enhance the crosslinking of CR and SBS copolymer, resulting in an improved high-, intermediate-, and low-temperature performance of desulfurized CR/SBS composite modified asphalt. Burgers’ model was found to be apposite in simulating the creep stages obtained from MSCR tests for CR/SBS composite modified asphalts. The superior high-temperature performance of desulfurized CR/SBS modified asphalt prepared with 4% SBS, 20% desulfurized rubber, and a force chemical reactor time of 45 min contributes to the good high-temperature elastic properties of the asphalt. Therefore, this combination is recommended as an optimal preparation process. In summary, the desulfurization of crumb rubber and using the force chemical reactor technique are beneficial to composite asphalt performance and can provide a new way of utilizing waste tire rubber.

## 1. Introduction

With the rapid and large-scale construction and maintenance of highways, the demand for high-performance asphalt binder has increased sharply over the past several years. Fatigue, rutting, and other diseases of asphalt pavement are closely related to the elastic and viscous deformation behaviors of asphalt under various working conditions [1,2]. At low temperatures, elasticity dominates, and asphalt behaves as an elastomer. As the ambient temperature drops, the asphalt pavement shrinks, which can generate cracking due to the increased stiffness of asphalt binder limiting this shrinkage [3,4]. Whereas during construction and at high temperatures, viscous properties dominate, and it behaves as a fluid. When the traffic load is applied to the asphalt pavement, the asphalt flows and resulting in unstable rutting.

Traditional asphalt does not have sufficient rheological properties to alleviate the issues mentioned above. To meet the performance demands of bituminous materials caused by the rapid increase in traffic volumes, a series of studies by various researchers have been conducted in search of innovative asphalt preparation methods. One of the effective methods is to use various modifiers to decrease the stiffness modulus of asphalt at low temperatures and increase the complex modulus at high temperatures. Therefore, modified asphalt is being vigorously developed as an alternative road construction material [5]. Styrene–butadiene–styrene (SBS) copolymers are the most common modifiers used to enhance the performance and extend the service life of asphalt pavements [6,7]. Compared with traditional asphalt, SBS-modified asphalts exhibit better high- and low-temperature performance owing to three-dimensional crosslinking networks formed by SBS copolymer. However, the high cost of SBS can significantly increase construction costs when large quantities are required. In recent years, researchers have gradually paid more attention to waste rubber, which is responsible for serious environmental pollution. Many researchers have investigated the potential use of crumb rubber (CR) from waste tires in modified asphalt. Compared with SBS-modified asphalts, large yields of crumb rubber (CR) modified asphalts can be produced at relatively low cost. Moreover, dispersed CR can enhance the mechanical and rheological properties of asphalt, specifically by reducing fatigue and low-temperature cracking. Nevertheless, the high viscosity and limited high-temperature performance of CR modified asphalts has restricted their widespread use.

To take full advantage of SBS and CR modified asphalt and to compensate for their respective weaknesses, SBS copolymer has been incorporated in CR modified asphalt [8,9]. Zhang et al. [10] showed that SBS copolymer is an effective physical modifier for enhancing the high-temperature stability of conventional CR modified asphalt, mainly due to the interlaced copolymer network structure formed between SBS and CR. Liang et al. [11] explored the effect of SBS content on the rheological properties of CR/SBS modified asphalt and found that as a composite modifier, CR and SBS copolymer can improve the deformation resistance of asphalt under various ambient temperatures. However, CR/SBS modified asphalt has some drawbacks; in particular, its low storage stability and high viscosity. The desulfurization of CR was shown to solve these problems [12]. Wang et al. [13] proposed the desulfurization of CR to break the stable crosslinking network structure, resulting in decreased viscosity and increased elasticity. The chain scission in rubber is responsible for improving the storage stability and processing ability of modified asphalt [14].

However, conventional prepared methods of desulfurized CR/SBS modified asphalt are generally the same as that of CR/SBS modified asphalt—that is, mixing SBS and CR with asphalt successively and shearing at high temperature and high speed. Therefore, few chemical reactions occurred in the modification process, which would mitigate the modifications effect. Researchers [15,16] found that external energy can overcome the nonpolar characteristic of CR that limits its dispersibility in asphalt, thereby improving the incompatibility problem of asphalt.

Based on the considerations above, this study used desulfurized CR and SBS polymer to modify rubber asphalt. A type of desulfurized CR/SBS modifier with a force chemical preparation method was proposed. Conventional tests, bending beam rheometer (BBR) tests, and DSR tests were performed to acquire the rheological properties of desulfurized CR/SBS composite modified asphalt at different frequencies and temperatures. Furthermore, two existing rheological models were conducted to derive the nonlinear viscoelastic parameters and high-temperature performance of the asphalts.

## 2. Materials and Methods

### 2.1. Materials

Pen-70 asphalt was used as the base asphalt for modification. The CR (#40 mesh), desulfurized CR, and SBS copolymer (LG 501, which is linear and produced in Korea) were provided by Jiangsu Zhonghong Environment Technology Co, Ltd. (Nanjing, China). Desulfurized CR/SBS composite modifiers with desulfurized CR (20% and 25% by weight of base asphalt binder) and 4% SBS copolymer were pre-prepared in a force chemical reactor with mixing times of 15 min, 30 min, and 45 min.

### 2.2. Preparation of Asphalt Samples

**Figure 1:**
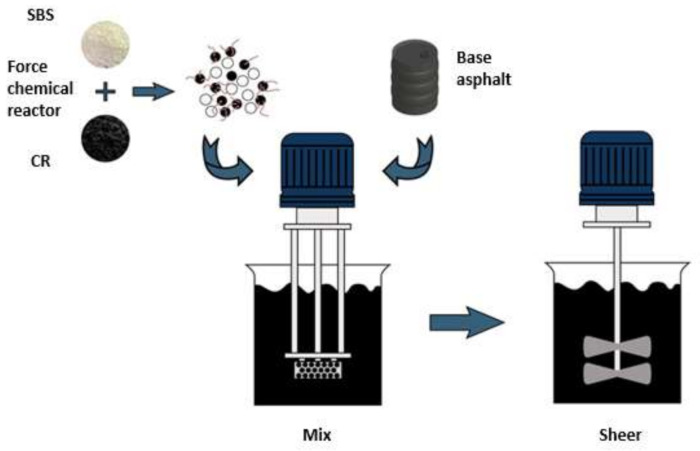
Flowchart of material preparation.

Desulfurized CR/SBS composite modified asphalt was prepared in a laboratory at 180 °C. First, the desulfurized CR/SBS composite modifier was added to the base asphalt and the blend was sheared at a speed of 6000 r/min for 1.5 h. Then, the speed was reduced to 1500 r/min for 1 h until fully swelled. The preparation process of the desulfurized CR/SBS composite modified asphalt is illustrated in Figure 1. Conventional CR/SBS composite modified asphalt with 20% CR and 4% SBS was prepared using the same method. Six types of asphalt were used in this study, as listed in Table 1.

### 2.3. Laboratory Tests

Prior to testing, all asphalt binders underwent short-term aging using the rolling thin film oven test (RTFOT) and were further aged in a pressure aging vessel (PAV) for 20 h to simulate aging. Rheological tests were repeated three times in each temperature range to obtain reliable results.

#### 2.3.1. Bending Beam Rheometer Test

The BBR test was performed on five PAV-aged asphalt samples at three different temperatures (−18 °C, −24 °C, and −30 °C), according to the AASHTO M320 standard. The stiffness modulus (S) and creep rate (m) were obtained and used to evaluate the low-temperature rheological characteristics of the asphalts. Here, the stiffness modulus characterizes the ability to resist deformation under constant load, and the m value describes the stress relaxation ability and sensitivity of stiffness to time. A larger S value and smaller m indicate poorer anti-cracking properties of asphalt.

#### 2.3.2. Linear Amplitude Sweep Test

The linear amplitude sweep test was performed on PAV-aged asphalt samples at 25 °C using an 8 mm dynamic shear rheometer (DSR, Anton Paar, Germany), according to the AASHTO TP101-12 [17] standard. First, a frequency sweep was performed at 0.1% strain in a frequency range of 0.2 to 30 Hz. The data were used to derive the slope of the relationship between frequency and shear modulus. Next, a linear amplitude sweep was carried out at a frequency of 10 Hz, with a linearly increasing strain amplitude from 0.05% to 30%. Then, the viscoelastic continuum damage (VECD) model was applied to obtain the damage characteristic and fatigue life curves at each strain amplitude. The fatigue damage can be characterized by damage intensity (D) and damage ratio C. When D is given, a smaller C indicates more serious damage. According to AASHTO TP 101-12, D = 0.35 was selected as the fatigue criterion.

#### 2.3.3. Multiple Stress Creep Recovery Test

The MSCR test was performed on RTFOT-aged asphalt samples using a 25 mm DSR geometry according to AASHTO T350-14 [18]. The specimen was loaded at two stress levels of 0.1 kPa and 3.2 kPa for twenty cycles and ten cycles, respectively. Each cycle consisted of 1 s of creep stage, followed by 9 s of recovery stage for a total of 10 s. The temperature range was 70–82 °C. The creep recovery rate (R) and non-recoverable creep compliance (J_nr_) were obtained and used to evaluate anti-rutting performance.

### 2.4. Rheological Models for Characterizing Asphalt Binders

#### 2.4.1. Burgers’ Model

One of the limitations of desulfurized CR/SBS composite modified asphalt is its poor high-temperature performance [13]. To further characterize the asphalt binder in the high-temperature range, this study investigated changes in nonlinear viscoelastic parameters. Taking the conventional CR/SBS composite modified asphalt as a control, creep curves from the MSCR test were fitted to Burgers’ model to obtain the viscoelastic parameters [19].

(1) $$\begin{array}{l} \varepsilon_{t}=\varepsilon_{e}+\varepsilon_{v}+\varepsilon_{d}\\ =\sigma \left[\frac{1}{E}+\frac{1}{\eta_{1}}t+\frac{1}{E_{2}}(1-e^{-\frac{E_{2}}{\eta_{2}}t})\right] \end{array}$$

(2) $$\varepsilon (t)=\sigma \left[\frac{1}{E_{1}}+\frac{1}{\eta_{1}}t+\frac{1}{E_{2}}(1-e^{-\frac{E_{2}}{\eta_{2}}t})\right]{\;(t<t}_{1})\;$$

(3) $$\varepsilon (t)=\sigma \left[\frac{t_{1}}{\eta_{2}}+\frac{1}{E_{2}}(1-e^{-\frac{E_{2}}{\eta_{2}}t_{1}})e^{-\frac{E_{2}}{\eta_{2}}(t-t_{1})}\right]\;(t\geq t_{1})\;$$

(4) $$AEE=\frac{1}{N}\sum_{1}^{N}\frac{\varepsilon_{predicted}(t_{i})-\varepsilon_{measured}(t_{i})}{\varepsilon_{measured}(t_{i})}$$

where *ε_e_* is the instantaneous elastic strain, *ε_v_* is the viscous flow, and *ε_d_* is the viscoelastic strain when the creep stress (*σ*) is applied; *E*_1_, *E*_2_, *η*_1_, and *η*_2_ are the four model parameters represented, respectively.

Liu [20] proposed a method for determining the four Burgers’ model parameters based on the constitutive equation of Burgers’ model, as presented in Equation (1). First, the time at the end of creep load (t = t_1_) is taken as the boundary, and the strain response in the creep section is expressed using Equation (2). The strain response in the recovery section is expressed by Equation (3). The initial viscous strain is taken as the measured strain at the end of a recovery cycle. The model parameters are obtained by fitting the recovery data and initial viscous strain to Equations (2) and (3). Finally, average absolute errors are calculated to evaluate the fitted results.

#### 2.4.2. Kelvin–Voigt Model

However, the burgers parameters were obtained mainly based on the creep stage of the MSCR test within a certain period. A recent study by Prashant et al. [21] proposed that three components (linear viscoelastic, nonlinear viscoelastic, and permanent strain) from the entire creep and recovery curve would help to better understand the behavior of modified binders. To verify the accuracy of Burgers’ model fitting results and improve the rigor of this research, the nonlinear viscoelastic characterization of the MSCR curve was performed.

The specified procedure is briefly described as follows:

Step 1: Hypothesis: The strain data of 10 cycles at 0.1 kPa were the same, ignoring the elastic component A.

Step 2: Determine the linear viscoelastic parameters.

Fit the recovery strain data of cycle number one at 0.1 kPa using Equation (5) to obtain the calculated linear parameters *B*, *C*, *D*, and *E*.

(5) $$\varepsilon \left(t\right)=0.1\left[A+B\left(1-e^{\frac{-t}{C}}\right)+D\left(1-e^{\frac{-t}{E}}\right)\right]-0.1\left[A+B\left(1-e^{\frac{-(t-1)}{C}}\right)+D\left(1-e^{\frac{-(t-1)}{E}}\right)\right]$$

Step 3: Determine the nonlinear viscoelastic parameters.

To determine the nonlinear viscoelastic parameter *G*_1_, *G*_2_, and *G*_3_, 1 to 10 s strain data points of the recovery stage at 3.2 kPa was analyzed. The parameter *G*_1_ is derived from fitting the first 10 data points (1 to 2 s) using Equation (6):

(6) $$\varepsilon {\left(t\right)}_{NLVE-G_{1}}=G_{1}\left[B\left(1-e^{\frac{-t}{C}}\right)+D\left(1-e^{\frac{-t}{E}}\right)\right]-\left[B\left(1-e^{\frac{-(t-1)}{C}}\right)+D\left(1-e^{\frac{-(t-1)}{E}}\right)\right]$$

Parameters *G*_2_ and *G*_3_ are derived from fitting the remaining 80 data points (2 to 10 s) with Equation (7):

(7) $$\varepsilon {\left(t\right)}_{NLVE-G_{2}-G_{3}}=G_{2}\left[3.2\left(J_{LVE\left(t=2\right)}-J_{LVE\left(t=1\right)}\right)\right]+G_{3}\left\{3.2\left[B\left(1-e^{\frac{-t}{C}}\right)+D\left(1-e^{\frac{-t}{E}}\right)\right]-\left[B\left(1-e^{\frac{-\left(t-1\right)}{C}}\right)+D\left(1-e^{\frac{-\left(t-1\right)}{E}}\right)\right]\right\}$$

where $J_{LVE(t=i)}=B\left(1-e^{\frac{-i}{C}}\right)+D\left(1-e^{\frac{-i}{E}}\right)$, *i* from 1 to 2.

Step 4: Calculate permanent strain.

(8) $$P\mathrm{er}manent\;\mathrm{Strain},\%=100{\left\{\left(Measured\;Strain\right)-\left(Calculated\;Strain\right)\right\}}_{at\;10}$$

## 3. Results and Discussion

### 3.1. Traditional Performance Test

Different modifiers and production technologies lead to complex physicochemical processes involving volatilization, oxidation, and condensation. Accordingly, the physical properties of asphalt including the softening point, penetration, and ductility will change. Three tests were carried out on each sample according to the relevant specifications. The results are presented in Table 2.

Compared with 70# base asphalt, the ductility and softening point of the composite modified asphalts were greatly improved, as expected for this type of material. Rubber chains released from the destroyed CR are inserted into the polymer SBS crosslinking network, which improves the crosslinking effect. At the same time, the low flow capacity of rubber restricts the asphalt flow. Both effects resulted in a high softening point and higher ductility. However, the penetration degree decreased. Previously, both CR and SBS copolymer were shown to selectively absorb the light fraction of the base asphalt during the swelling process, resulting in an increase in asphaltene and resin content [22,23]. While asphaltene and resin have a hardening effect on asphalt, the hardening effect was also enhanced with increasing CR/SBS composite modifier dosage and swelling degree. In general, the M series modified asphalts had higher ductility and softening points than J series modified asphalts, indicating that both pre-preparation in a force chemical reactor and desulfurized CR can improve the high and low temperature performance. However, increasing the dosage of desulfurized CR to 25% reduced its penetration and ductility at 5 °C.

**Table 2:** Technical indexes of test samples.

Test Index	70#	J-20	M-20	M-25	M-15MINS	M-45MINS
25 °C Penetration/mm	61	57.5	56	55.43	55.67	58.7
5 °C Ductility/cm	25	38.2	59.6	56.3	57.8	62
Softening point/°C	54.8	65.1	80.75	86.5	75.5	87.1

### 3.2. Rheological Properties

Traditional performance tests can characterize asphalt performance from a macro perspective at a given temperature but fail to reflect the microstructural changes. In contrast, characterizing desulfurized CR/SBS composite modified asphalt based on rheological properties can effectively bridge the gap between the microstructure and macroscopic motion. Three rheological tests were conducted to evaluate the effect of desulfurized CR and pre-preparation time of desulfurized CR/SBS composite modifiers in a force chemical reactor on the rheological properties of modified asphalt.

#### 3.2.1. Bending Beam Rheometer Test Results

**Figure 2:**
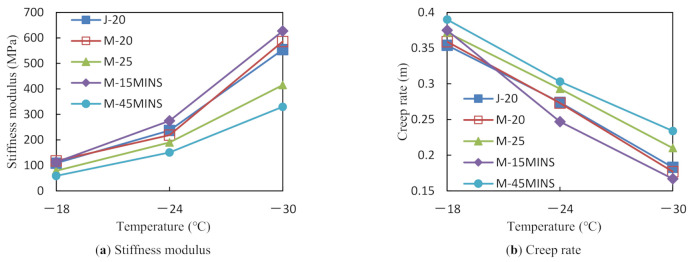
Bending beam rheometer (BBR) test results.

Figure 2 presents the evolution of stiffness modulus (S) and creep rate (m) with loading time for TFOT-PAV aged CR/SBS modified asphalts at −18, −24, and −30 °C. As the temperature decreased, S values of the five modified asphalts increased; however, the creep rate (M) exhibited a decreasing trend. When the temperature dropped to −30 °C, all samples reached the failure point. This could be because as the temperature drops, the elastic component in the asphalt increases gradually, and the asphalt becomes harder and more brittle, thereby reducing its low-temperature relaxation ability [22]. At −30 °C, the asphalt has already exceeded its low-temperature limit. It is worth noting that the S and m values of the M-25 and M-45MINS modified asphalts are significantly better than those of the other modified asphalts. This may be because the high resilience and viscoelasticity of rubber can facilitate low-temperature deformation of the rubber asphalt. The increase in CR content resulted in greater elastic deformation. However, the test data also revealed an oddity. The S value at a certain temperature is not higher than 300 MPa, while the m value is lower than 0.3, suggesting that the conclusions made from S and m values as indexes are not sufficiently convincing.

As in previous studies [24,25], the S and m values were fitted in the semi-logarithmic and arithmetic coordinate systems (T_L,S_, T_L,m_) to obtain the low-temperature grade temperature (T_LG_) of the modified asphalts, as shown in Table 3. The T_LG_ value indicates the low-temperature crack resistance, with a lower T_LG_ indicating superior crack resistance. The T_LG_ value is higher than −30 °C, which is consistent with the failure points of all the asphalt samples of above −30 °C within the tested temperature range. Therefore, the T_LG_ of the BBR test can be used as an index for evaluating the low-temperature crack resistance of desulfurized CR/SBS composite modified asphalts. The T_LG_ values of the five asphalts can be ordered as follows: M-45MINS<M-25<M-20<J-20<M-15MINS. The results show that the desulfurization of CR, increase in CR content, and application of the force chemical reactor technique can improve the low-temperature crack resistance of modified asphalts.

#### 3.2.2. Linear Amplitude Sweep Test Results

**Figure 3:**
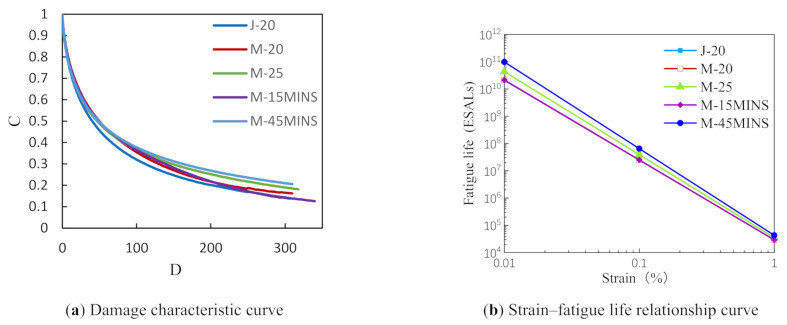
Linear amplitude sweep (LAS) test results.

Damage characteristic curves of the asphalt samples are shown in Figure 3. The damage degrees of the M-30MINS and M-45MINS modified asphalts were similar when C = 0.35, whereas M-30MINS appears to have better integrity than M-45MINS when C > 0.35. In addition, the damage rate of the M-20 and M-25 modified asphalts are similar, indicating that CR content has little effect on the fatigue performance of desulfurized CR/SBS composite modified asphalt.

The LAS test data were further processed using the viscoelastic continuum damage (VECD) model to obtain the relationship between strain and fatigue life. As shown in Figure 2b, the fatigue life of asphalt decreases with increasing strain and reached 10^11^ ESALs under the smallest strain. The effects on fatigue life may be due to the addition of carbon black to rubber, which can improve the aging resistance of asphalt. The fatigue resistance of M-45MINS modified asphalt was consistently superior to those of other modified asphalts, and an entire order of magnitude higher than the J-20 modified asphalt at low strain levels. The change in microstructure of CR as a result of the desulfurization process, which breaks the internal sulfur bonds resulting in an increase in rubber transferase activity, makes it easier to absorb the light components of the asphalt [13,14]. At the same time, increasing time in the force chemical reactor may cause devulcanized CR to release more light rubber molecules, which re-crosslink with SBS in the asphalt, thereby improving the fatigue resistance of the asphalt.

#### 3.2.3. Multiple Stress Creep Recovery Test Results

**Figure 4:**
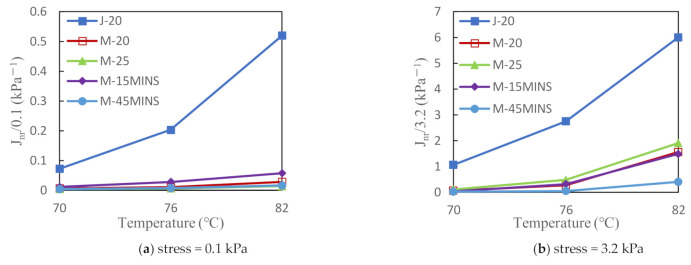
Influence of temperature on unrecovered strain (J_nr_) of asphalt binder at different stress levels.

**Figure 5:**
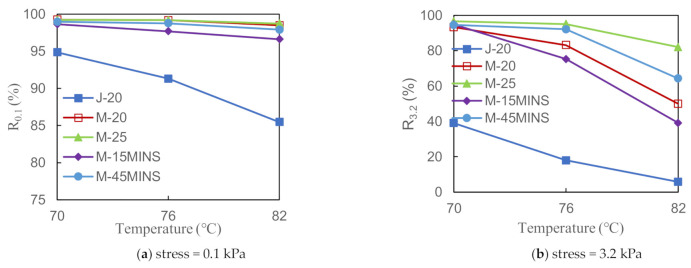
Influence of temperature on recovered strain (R) of asphalt binder at different stress levels.

Figure 4 and Figure 5 show the MSCR test results for five composite modified asphalts at different temperatures. A lower J_nr_ value indicates greater resistance to permanent deformation as well as better performance against rutting. Figure 4 shows that the J_nr_ value of the composite modified asphalt gradually increased with increasing temperature at both stress levels. However, at 3.2 kPa, the J_nr_ of J-20 modified asphalt was significantly higher than that of the M series modified asphalts. Under high stress, the creep recovery ability of the desulfurized CR/SBS composite modified asphalts was better than that of the conventional CR composite modified asphalt, which was likely due to the desulfurization of CR. The desulfurization process can strengthen the molecular network structure of CR, and consequently improve the high-temperature stability of asphalt to a certain extent [26]. The J_nr_ value did not noticeably change among the same M series modified asphalts at 0.1 kPa. However, the J_nr_ values of the M-25 and M-45MINS modified asphalts significantly deviated at 3.2 kPa stress and 82 °C. In summary, modified asphalt is not sensitive to temperature and CR content at low stress levels; however, an appropriate increase in CR content and force chemical reactor time can significantly improve the deformation resistance under high temperature and high stress. Increasing the reactor time promotes chain fracture in rubber and decreases the degree of crosslinking, leading to the swelling of lighter components into CR.

Furthermore, higher R values of binders indicate better strain recovering ability. From Figure 5, the curve of J-20 modified asphalt is significantly different from that of the M series modified asphalts, and its R value is lower. This also indicates that the crosslinking network structures formed by CR and between CR and SBS copolymer can complement each other to comprehensively improve the asphalt performance. At low stress, changes in temperature and CR content have little effect on the R value of M series modified asphalts, which also indicates that desulfurized CR/SBS composite-modified asphalt is not sensitive to temperature and CR content at low stress levels, and its high-temperature stability is excellent. Under high stress, the increase in temperature significantly reduces the R value, whereas M-20 modified asphalt showed better creep recovery. This may be due to the increase in viscosity and weakened resistance to deformation caused by the high stress and high temperature. Since the modulus of rubber is large and the asphalt’s resistance to deformation is mainly provided by the CR, as the CR content increases, it can more effectively preserve the elastic properties.

### 3.3. Viscoelasticity Analysis Based on Multiple Stress Creep Recovery Test

#### 3.3.1. Burgers’ Model Fitting Results

**Figure 6:**
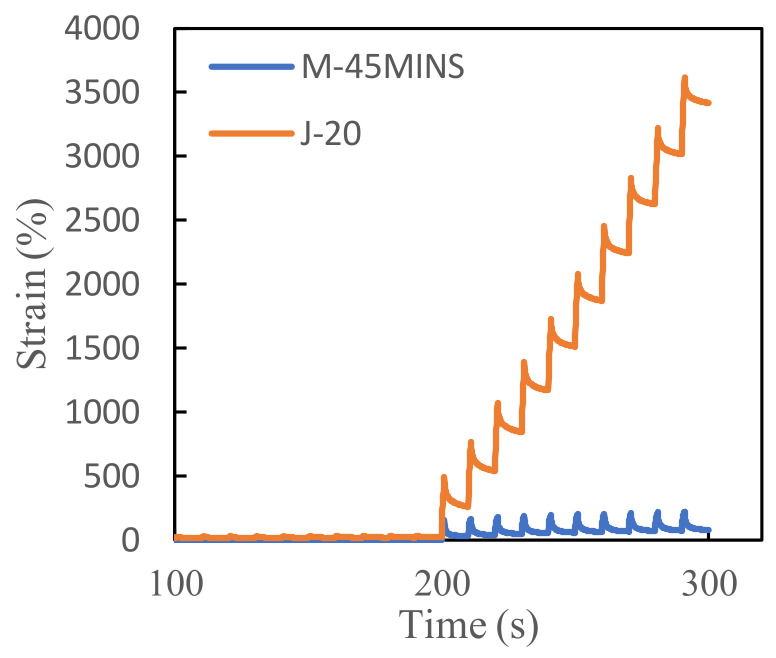
Typical creep and recovery cycle during multiple stress creep recovery (MSCR) test.

According to the MSCR test results, the high-temperature stability of M-45MINS modified asphalt is better than the other modified asphalts. For brevity, only the creep recovery data of the 10th cycle of the M-45MINS modified asphalt were analyzed, and J-20 modified asphalt was used as a control, as shown in Figure 6.

Table 4 shows the average absolute error (AAE) values of the MSCR test data at two loading levels fitted to Burgers’ model. Burgers’ model fits better with the creep stage of conventional CR and desulfurized CR/SBS composite modified asphalts at high temperature, with an AAE below 10%. However, the J-20 modified asphalt fits Burgers’ model significantly better than M-45MINS modified asphalt [27]. Even at high stress and high temperature, the AAE value of the J-20 modified asphalt was approximately zero. This may be explained by the technical limitations of the method and the intrinsic limitations of the model, such as the number of spring and dashpot elements and the presence of only relaxation time in the equation [28].

The Burgers’ model parameters in Table 5 suggest that an increase in temperature and stress level reduces the four model parameter values, which is manifested as a decrease in the high-temperature stability of the asphalt. Under the same stress level and temperature, the instantaneous elastic modulus (E_1_) of desulfurized CR/SBS composite modified asphalt is significantly higher than that of conventional CR/SBS composite modified asphalt. Thus, the desulfurized CR can restore some elasticity and improve the deformation resistance of modified asphalt under instantaneous loading. Out of the four Burgers’ model parameters, those characterizing the non-recoverable permanent deformation (η_1_) of asphalt are most important, because viscous flow has a large impact on asphalt binder performance. A higher η_1_ value indicates better anti-rutting properties. In general, η_1_ is found to decrease with increasing temperature [29]. The η_1_ value of M-45MINS is higher than that of the J-20 modified asphalt, indicating that the high-temperature performance of M-45MINS modified asphalt is better. At low stress levels, the η_1_ values of the two asphalts change very little, but at high stress levels, they decrease significantly with increasing temperature. This finding is consistent with the variation of non-recoverable creep compliance observed in Figure 4.

#### 3.3.2. Kelvin–Voigt Model Fitting Results

Table 6 shows the linear viscoelastic parameters (B, C, D, E), nonlinear viscoelastic parameters (G_1_, G_2_, G_3_), and PS for two types of asphalt.

Nonlinear viscoelastic parameters can be used to characterize the recovery behavior for asphalt [20]. Parameter G_1_ represents the vertical shift of the recovery curve from 1 to 2 s. The G_1_ value of M-45MINS modified asphalt is larger than that of J-20 modified asphalt, indicating that M-45MINS modified asphalt has better deformation recovery under instantaneous loading. It is consistent with the change of E_1_ value of Burgers’ model, since desulfurized CR restores elasticity. Parameters G_2_ and G_3_ represent the vertical shift and slope changes of the recovery curve from 2 to 10 s. The derived G_3_ value of two asphalts are negative and increase closely to zero. The fitting variation of G_3_ accurately reflects the change of recovery curve, indicating that the Kelvin–Voigt model can be used to fit the viscoelastic mechanism of CR modified asphalt. As the recovery time increases, the delayed elastic deformation of asphalt gradually recovered completely. With the increase of temperature, the PS value of M-45MINS asphalts increase sharply, indicating that high temperature impairs the asphalt’s ability to resist deformation. Meanwhile, when the PS value of J-20 asphalt under 82 °C decreases, it may due to the fitting errors.

**Table 6:** Viscoelastic parameters and PS.

Parameters	M-45MINS			J-20		
	70 °C	76 °C	82 °C	70 °C	76 °C	82 °C
B	0.04188	0.06221	0.08895	0.36835	0.60998	0.84986
C	0.58801	0.60414	0.62757	1.01302	1.02523	0.98769
D	2.51056	3.8495	9.94901	59.20465	187.36224	514.839
E	18.41396	18.29115	24.90884	32.00404	46.03671	65.2637
G_1_	1.03109	0.74513	0.75977	0.52863	0.50518	0.57031
G_2_	0.67731	0.44075	0.4745	0.33085	0.31722	0.36266
G_3_	−0.11469	−0.11445	−0.08411	−0.02759	−0.01106	−0.0026
PS	3.20959	4.5752	6.36363	8.36741	8.83956	4.97893

## 4. Conclusions

A type of desulfurized CR/SBS modifier with a force chemical preparation method was proposed. The rheological properties of desulfurized CR/SBS composite modified asphalt were investigated through rheological tests and rheological models. The conclusions can be summarized as follows:

1. As a result of the CR chain inserted into the SBS crosslinking network and the low flow capacity of rubber, all of the modified asphalts were found to have higher ductility and softening points than the base asphalt. The force chemical reactor may strengthen this crosslinking effect. However, both CR and SBS copolymer were shown to selectively absorb the light fraction of the base asphalt, resulting in the decrease of penetration degree.
2. The BBR test showed that the high resilience and viscoelasticity of rubber facilitates low-temperature deformation of CR/SBS composite modified asphalt. Desulfurized CR/SBS modifier and its force chemical preparation method were found to be more effective than conventional CR/SBS modifier in improving the asphalt behavior at low temperatures. T_LG_ can be used as an index for evaluating the low-temperature crack resistance of desulfurized CR/SBS composite modified asphalts.
3. The results of LAS tests indicated that both the desulfurization of CR and force chemical reactor time successfully enhanced the fatigue resistance of asphalt, because the change in the microstructure of CR makes it absorb more light components, which is beneficial for resisting fatigue disease. The fatigue resistance of M-45MINS modified asphalt was superior to those of other modified asphalts.
4. Comparing the MSCR tests in different temperatures and at different addition levels of CR, the desulfurization of CR and force chemical reactor time mitigate the J_nr_ and R values. However, modified asphalt is not sensitive to temperature and CR content at low stress levels.
5. Rheological modeling of the creep-recovery behavior of desulfurized CR/SBS composite modified asphalt suggests that an increase in both temperature and stress level significantly affect the four Burgers’ model parameters. Consequently, the high-temperature stability of the asphalt decreases. In addition, the nonlinear viscoelastic characterization of the MSCR curve based on the Kelvin–Voigt model verify the Burgers’ model fitting results’ accuracy.

## Figures and Tables

**Table 1:** Types of asphalt samples.

Asphalt Type	Proportion of Modifier	Force Chemical Reactor Time
70#	-	-
J-20	4% SBS + 20% rubber	-
M-20	4% SBS + 20% desulfurized rubber	30 min
M-25	4% SBS + 25% desulfurized rubber	30 min
M-15MINS	4% SBS + 20% desulfurized rubber	15 min
M-45MINS	4% SBS + 20% desulfurized rubber	45 min

**Table 3:** Low-temperature grade temperature (T_LG_) of asphalt samples.

Asphalt Type	J-20	M-20	M-25	M-15MINS	M-45MINS
$T_{L,s}$	−25.58	−24.73	−24.32	−24.77	−25.43
$T_{L,m}$	−21.44	−21.49	−22.84	−21.23	−23.97
$T_{\mathrm{LG}}$	−21.44	−21.49	−22.84	−21.23	−23.97

**Table 4:** Average absolute error value for fitted multiple stress creep recovery (MSCR) test data.

Items	Stress	Temperature		
		70 °C	76 °C	82 °C
M-45MINS	0.1 kPa	8.49%	7.33%	6.26%
	3.2 kPa	8.12%	6.36%	0.68%
J-20	0.1 kPa	2.16%	0.14%	0.02%
	3.2 kPa	0.01%	0	0

**Table 5:** Burgers’ model parameters.

Stress	Temperature	M-45MINS				J-20			
		E_1_	E_2_	η_1_	η_2_	E_1_	E_2_	η_1_	η_2_
0.1 kPa	70 °C	36.06	16.22	30.37	71.33	4.41	3.16	7.17	28.67
	76 °C	32.01	16.40	2.18	60.55	2.68	1.99	4.5	22.02
	82 °C	13.58	7.94	15.85	59.45	1.37	1.37	3.09	20.77
3.2 kPa	70 °C	24.69	13.06	24.90	51.23	1.02	7.83	16.78	8.33
	76 °C	14.26	8.61	17.70	33.24	0.40	10.93	21.84	3.28
	82 °C	2.60	8.55	17.06	19.67	0.18	16.28	30.50	1.53

## Data Availability

Data is contained within the article.

## References

[1] Wang L., Chang C. (2015). Rheological Evaluation of Polymer Modified Asphalt Binders. J. Wuhan Univ. Technol. Mater. Sci. Ed..

[2] Fernandes M.R., Forte M.M., Leite L.F. (2008). Rheological Evaluation of Polymer-Modified Asphalt Binders. Mater. Res. Ibero Am. J. Mater..

[3] Hassanpour-Kasanagh S., Ahmedzade P., Fainleib A.M., Behnood A. (2020). Rheological properties of asphalt binders modified with recycled materials: A comparison with Styrene-Butadiene-Styrene (SBS). Constr. Build. Mater..

[4] Behnood A., Olek J. (2017). Rheological properties of asphalt binders modified with styrene-butadiene-styrene (SBS), ground tire rubber (GTR), or polyphosphoric acid (PPA). Constr. Build. Mater..

[5] Wang L., Razaqpur G., Xing Y., Chen G. (2015). Microstructure and rheological properties of aged and unaged polymer-modified asphalt binders. Road Mater. Pavement Des..

[6] Lo Presti D. (2013). Recycled Tyre Rubber Modified Bitumens for road asphalt mixtures: A literature review. Constr. Build. Mater..

[7] Bamigboye G.O., Bassey D.E., Olukanni D.O., Ngene B.U., Adegoke D., Odetoyan A.O., Kareem M.A., Enabulele D.O., Nworgu A.T. (2021). Waste materials in highway applications: An overview on generation and utilization implications on sustainability. J. Clean. Prod..

[8] Kok B.V., Colak H. (2011). Laboratory comparison of the crumb-rubber and SBS modified bitumen and hot mix asphalt. Constr. Build. Mater..

[9] Dong F., Yu X., Liu S., Wei J. (2016). Rheological behaviors and microstructure of SBS/CR composite modified hard asphalt. Constr. Build. Mater..

[10] Zhang F., Hu C. (2015). The research for structural characteristics and modification mechanism of crumb rubber compound modified asphalts. Constr. Build. Mater..

[11] Liang M., Xin X., Fan W., Luo H., Wang X., Xing B. (2015). Investigation of the rheological properties and storage stability of CR/SBS modified asphalt. Constr. Build. Mater..

[12] Sienkiewicz M., Borzędowska-Labuda K., Wojtkiewicz A., Janik H. (2017). Development of methods improving storage stability of bitumen modified with ground tire rubber: A review. Fuel Process. Technol..

[13] Rasool R.T., Song P., Wang S. (2018). Thermal analysis on the interactions among asphalt modified with SBS and different degraded tire rubber. Constr. Build. Mater..

[14] Song P., Zhao X., Cheng X., Li S., Wang S. (2018). Recycling the nanostructured carbon from waste tires. Compos. Commun..

[15] Fu Q., Xu G., Chen X., Zhou J., Sun F. (2019). Rheological properties of SBS/CR-C composite modified asphalt binders in different aging conditions. Constr. Build. Mater..

[16] Attia M., Abdelrahman M. (2009). Enhancing the performance of crumb rubber-modified binders through varying the interaction conditions. Int. J. Pavement Eng..

[17] American Association of State and Highway Transportation Officials (2018). AASHTO TP 101-12 (2018) Standard Method of Test for Estimating Fatigue Resistance of Asphalt Binders Using the Linear Amplitude Sweep.

[18] American Association of State and Highway Transportation Officials (2018). AASHTO T 350-14 (2018) Standard Method of Test for Multiple Stress Creep Recovery (MSCR) Test of Asphalt Binder Using a Dynamic Shear Rheometer (DSR).

[19] Liu H., Zeiada W., Al-Khateeb G.G., Shanableh A., Samarai M. (2021). Use of the multiple stress creep recovery (MSCR) test to characterize the rutting potential of asphalt binders: A literature review. Constr. Build. Mater..

[20] Liu Y., You Z. (2008). Determining Burger’s Model Parameters of Asphalt Materials Using Creep-Recovery Testing Data. Proceedings of the Symposium on Pavement Mechanics and Materials at the Inaugural International Conference of the Engineering Mechanics Institute-Pavements and Materials 2008: Modeling Testing, and Performance, Minneapolis, MN, USA, 18–21 May 2008.

[21] Shirodkar P., Mehta Y., Nolan A., Dahm K., Dusseau R., McCarthy L. (2012). Characterization of creep and recovery curve of polymer modified binder. Constr. Build. Mater..

[22] Dong D., Huang X., Li X., Zhang L. (2012). Swelling process of rubber in asphalt and its effect on the structure and properties of rubber and asphalt. Constr. Build. Mater..

[23] Xiang L., Cheng J., Que G. (2009). Microstructure and performance of crumb rubber modified asphalt. Constr. Build. Mater..

[24] Shen J., Arnirkhanian S., Tang B. (2007). Effects of rejuvenator on performance-based properties of rejuvenated asphalt binder and mixtures. Constr. Build. Mater..

[25] You Z., Mills-Beale J., Fini E., Goh S.W., Colbert B. (2011). Evaluation of Low-Temperature Binder Properties of Warm-Mix Asphalt, Extracted and Recovered RAP and RAS, and Bioasphalt. J. Mater. Civ. Eng..

[26] Yu H., Leng Z., Gao Z. (2016). Thermal analysis on the component interaction of asphalt binders modified with crumb rubber and warm mix additives. Constr. Build. Mater..

[27] Lagos-Varas M., Movilla-Quesada D., Arenas J.P., Raposeiras A.C., Castro-Fresno D., Calzada-Perez M.A., Vega-Zamanillo A., Maturana J. (2019). Study of the mechanical behavior of asphalt mixtures using fractional rheology to model their viscoelasticity. Constr. Build. Mater..

[28] Domingos M.D., Faxina A.L. (2015). Rheological behaviour of bitumens modified with PE and PPA at different MSCR creep-recovery times. Int. J. Pavement Eng..

[29] Kumar R., Saboo N., Kumar P., Chandra S. (2017). Effect of warm mix additives on creep and recovery response of conventional and polymer modified asphalt binders. Constr. Build. Mater..

